# Complex optical transport, dynamics, and rheology of intermediately attractive emulsions

**DOI:** 10.1038/s41598-023-28308-6

**Published:** 2023-01-31

**Authors:** Yixuan Xu, Thomas G. Mason

**Affiliations:** 1 Department of Materials Science and Engineering, University of California- Los Angeles, Los Angeles, CA 90095 USA; 2 Department of Chemistry and Biochemistry, University of California- Los Angeles, Los Angeles, CA 90095 USA; 3 Department of Physics and Astronomy, University of California- Los Angeles, Los Angeles, CA 90095 USA

**Keywords:** Physical chemistry, Chemical engineering, Soft materials, Optical spectroscopy, Chemical physics, Statistical physics

## Abstract

Introducing short-range attractions in Brownian systems of monodisperse colloidal spheres can substantially impact their structures and consequently their optical transport and rheological properties. Here, for size-fractionated colloidal emulsions, we show that imposing an intermediate strength of attraction, well above but not much larger than thermal energy ($\approx 5.6$ $k_{\textrm{B}}T)$, through micellar depletion leads to a striking notch in the measured inverse mean free path of optical transport, $1/\ell ^*$, as a function of droplet volume fraction, $\phi$. This notch, which appears between the hard-sphere glass transition, $\phi _{\textrm{g}}$, and maximal random jamming, $\phi _{\textrm{MRJ}}$, implies the existence of a greater population of compact dense clusters of droplets, as compared to tenuous networks of droplets in strongly attractive emulsion gels. We extend a prior decorated core-shell network model for strongly attractive colloidal systems to include dense non-percolating clusters that do not contribute to shear rigidity. By constraining this extended model using the measured $1/\ell ^*(\phi )$, we improve and expand the microrheological interpretation of diffusing wave spectroscopy (DWS) experiments made on attractive colloidal systems. Our measurements and modeling demonstrate richness and complexity in optical transport and shear rheological properties of dense, disordered colloidal systems having short-range intermediate attractions between moderately attractive glasses and strongly attractive gels.

## Introduction

Imposing short-range attractive interactions between colloids in a continuous liquid phase can dramatically alter a wide range of Brownian colloidal systems, both equilibrium and non-equilibrium, leading to different structural morphologies, dynamics, and physical properties^1–8^. In particular, for higher colloidal volume fractions, $\phi$, beyond the dilute limit and for attractive interactions that are much stronger than thermal energy, $k_{\textrm{B}}T$, where $k_{\textrm{B}}$ is Boltzmann’s constant and *T* is the temperature, networks of colloids can form, yielding colloidal gels^6,9–12^. The shape, size distribution, and deformability of the colloids, the history of preparation and flow imposed on the system, and the type, range, and strength of the intercolloidal attractions are all factors that can affect the structure, dynamics, and properties of colloidal gels^13,14^. For instance, short-range strongly attractive systems that are formed through slippery bonding^15,16^, arising from a deep secondary attractive well in the interaction potential, can have different distributions of local coordination numbers than solid-particulate systems that are formed through shear-rigid bonding^14–16^, arising from an extremely deep primary attractive well^1,17^. Strongly attractive colloidal gels represent one specific type of colloidal system having locally disordered structure, yet for which a characteristic length scale associated with an average mesh-size can emerge through the process of diffusion-limited cluster aggregation (DLCA)^2,18^. By contrast, in the other limit of very weak colloidal attractions, approaching nearly hard (NH) interactions, disordered colloidal glasses can be formed through rapid osmotic compression to dense $\phi$. Further osmotic compression of a colloidal glass to even higher $\phi$ can lead to a jammed colloidal glass.

Although colloidal gels and colloidal glasses are both disordered and can exhibit low-frequency plateau shear elasticity, they represent two different types of soft elastic systems that are distinguished primarily by the strength of colloidal attractions relative to $k_{\textrm{B}}T$. We refer to dense colloidal systems that have interactions ranging from weakly attractive to hard and that lack a long-time relaxation as colloidal glasses, since classical concepts of the ergodic-nonergodic transition for hard spheres apply in this limit of vanishing attractive strength. For $\phi$ just above the glass transition volume fraction of hard monodisperse spheres, $\phi _{\textrm{g}} \approx$ 0.56–0.58^19–21^, such hard-sphere glasses exhibit a zero-frequency elastic plateau shear storage modulus, $G^\prime _\text {p}$, as a consequence of nonergodicity and very limited accessible translational microstates per colloid on average; yet, the magnitude of $G^\prime _\text {p}$ remains finite even as $\phi$ is increased somewhat above $\phi _{\textrm{g}}$. For example, classic mode-coupling theory (MCT)^22,23^ describes glassy dynamics in glass-forming liquids as well as in hard-interacting colloidal systems^24^; MCT predicts a divergence in the relaxation time of density fluctuations as $\phi$ is raised towards $\phi _{\textrm{g}}$. However, for even larger $\phi$, colloidal glasses of hard rigid spheres approach the maximal random jamming (MRJ) point^25^, $\phi _{\textrm{MRJ}} \approx$ 0.646 (an insightful refinement of the earlier concept of random close packing^26^), the zero-frequency $G^\prime _\text {p}$ effectively diverges when the ideally rigid colloids jam and touch. By contrast, colloidal gels, which consist of space-filling networks, can have substantial zero-frequency $G^\prime _\text {p}$ for $\phi$ well below $\phi _{\textrm{g}}$. In both cases, if the colloidal objects are soft, rather than highly rigid solid spheres, this softness can modify the behavior, and $G^\prime _\text {p}$ does not diverge at $\phi _{\textrm{MRJ}}$^27^. In addition, the strengths of short-range attractions and also stabilizing repulsions, which may be present at even shorter range than the attractions, can influence both the onset and $\phi$-dependence of $G^\prime _\text {p}$^28,29^. For low enough $\phi$ and intermediate attractive strengths, two-phase coexistence between a gas-like monomer phase and a liquid-like non-percolating cluster phase can occur^6,30,31^; yet, for $\phi$ well below $\phi _{\textrm{g}}$, owing to the absence of shear-rigid percolating networks, the shear rheology of such gas-cluster systems is dominantly viscous, not elastic.

While prior simulations^32–35^, theories^36,37^, and experiments, such as dynamic light scattering (DLS)^38–43^ and three-dimensional (3D) high-resolution confocal microscopy^35,44–46^, have addressed various aspects of short-range attractive colloidal systems, interesting questions still remain. In particular, the connections between colloidal dynamics and structures to observable macroscopic properties, such as optical transport and rheological properties, have not been systematically explored experimentally and explained self-consistently for intermediate attractive strengths. This is particularly true regarding passive microrheological interpretations of complex dense colloidal systems having short-range slippery attractions. Recently, in a key advance, Kim et al.^47^ has shown how to correct diffusing wave spectroscopy (DWS)^48–51^ measurements of mean square displacements (MSDs) for collective scattering that occurs in dense, nearly hard interacting, size-fractionated, colloidal emulsions at high $\phi$ using measurements of the inverse scattering mean free path, $1/\ell ^*(\phi )$. This advance has yielded quantitative agreement between $G^\prime _\text {p}(\phi )$ measured using mechanical rheometry and $G^\prime _\text {p}(\phi )$ derived from DWS MSDs through the generalized Stokes-Einstein relation (GSER) of passive microrheology^52^. Using a modern form of DWS that is suitable for non-ergodic samples^53–58^ and that provides reproducible plateau MSDs and also performing collective scattering corrections, amounting to factors that can be well over 2 for dense $\phi$, are both extremely important in order to measure accurate DWS MSDs in dense elastic colloidal systems, irrespective of interactions. Thus, performing and interpreting the DWS experiments properly are both necessary for ensuring quantitatively accurate passive microrheology using the GSER.

Going beyond nearly hard interactions, this improved DWS technique has also been applied to size-fractionated colloidal emulsions that are subjected to short-range micellar depletion attractions. Kim et al.^59^ have explored the moderately attractive (MA) regime, where $|U_{\textrm{d}}| \approx$ 2–3 $k_{\textrm{B}}T$; by contrast, Xu et al.^60^ have investigated the strongly attractive (SA) regime, where $|U_{\textrm{d}}| \gg k_{\textrm{B}}T$ (*i.e.* specifically $|U_{\textrm{d}}| \approx$ 15 $k_{\textrm{B}}T$). For dense MA emulsions, the existence of excess MSDs at long times, related to hetereogeneous larger-scale motion of a minor subset of droplets in the system, have been observed and identified; yet, quantitative passive microrheological agreement can still be obtained for $G^\prime _\text {p}(\phi )$ using plateau MSDs at intermediate times, which accurately reflect the average motion of droplets in shear-stress bearing regions when interpreted using the GSER. At least over the limited range of dense $\phi$ explored, the measured $1/\ell ^*(\phi )$ of MA emulsions effectively equals that of emulsions having NH interactions^47^. By contrast, for dense SA emulsions, excess MSDs are effectively suppressed by the strong attractions, and the intermediate-time plateaus in MSDs extend to long times. However, the asymmetric shape of the $1/\ell ^*(\phi )$ for SA emulsions substantially differs from the highly symmetric shape of the $1/\ell ^*(\phi )$ associated with the same emulsion having NH interactions. Moreover, the meaning of the scattering probe associated with DWS MSDs requires a more sophisticated interpretation; rather than simply being a droplet, the DWS scattering probe in the strongly attractive limit has been identified to be effectively a local dense cluster (LDC) of droplets, which has an average size similar to a tetrahedral cluster. Therefore, the effective radius of the relevant DWS scattering probe, appropriate for use in the GSER, is increased by a $\phi$-independent factor of $\approx 2$. To arrive at this effective DWS probe-size and interpretation of an average DWS probe as approximately similar to tetrahedral cluster in this strongly slippery-attractive colloidal system, Xu et al. have introduced a decorated core-shell network (DCSN) model, which provides a self-consistent means of deducing the relative fractions of surface decorating droplets, shell droplets, and core-network droplets from the measured $1/\ell ^*(\phi )$. However, a colloidal emulsion system in the intermediately attractive (IA) regime, which lies between the moderately and strongly attractive regimes, has not yet been systematically investigated using this improved DWS technique. If performed, a new study on IA emulsions could potentially give rise to additional quantitative insights beyond an earlier DWS study on attractive polymer-emulsion systems^61^. In particular, this new DWS study could provide a $\phi$-dependent quantitative comparison of passive microrheological measurements, made optically, with macroscopic rheology measurements. Moreover, the results of this new DWS study could be compared directly to previously published studies of NH, MA, and SA emulsions.

Here, we show that passive DWS microrheology can be performed quantitatively on a dense IA emulsion system having a short-range, slippery interdroplet attraction ($\approx 5.6$ $k_{\textrm{B}}T$) induced by micellar depletion, provided that the substantial complexities in the DWS correlation functions are identified and interpreted without oversimplification. Our measurements of optical transport properties, DWS correlation functions, and macroscopic mechanical properties of dense IA emulsions cover a much larger range of $\phi$ than prior studies in which the dense emulsion systems were formed through gravitational creaming of larger droplets and in which different experimental techniques were used^12,62^. Our measurements reveal a $\phi$-dependent richness, both in optical transport properties and DWS MSDs, that has not been previously observed for either MA or SA emulsion systems. Most strikingly, the overall magnitude of $1/\ell ^*(\phi )$ for $\phi \le 0.65$ is reduced compared to SA emulsions, indicating a lower surface-to-volume ratio of attractive networks and constituent clusters, and a notch-like depression is also observed for $\phi _{\textrm{g}} \le \phi \le \phi _{\textrm{MRJ}}$. In addition to applying the essential MSD correction for collective light scattering in DWS^47^, we extract the true self-motion MSDs of scattering probes in shear-supporting regions of the attractive emulsion at intermediate times and disregard long-time excess MSDs that are known to arise from a small sub-population of droplets having higher mobility^59^. Moreover, we hypothesize that the scattering probes associated with DWS MSDs are neither solely single droplets throughout the entire range of $\phi$ as in MA emulsions^59^, nor solely LDCs of tetrahedra of droplets throughout the entire range of $\phi$ as in SA emulsions^60^. Instead, for IA emulsions, our measurements suggest that the effective average radius of the DWS probes is dominated by droplets below the cluster-jamming point at lower dense $\phi$, varies continuously through a transition region between droplets and tetrahedral LDCs for $\phi$ in the notch, and then is dominated by LDCs at higher dense $\phi$ above the cluster-jamming limit near $\phi _\text {MRJ}$. To quantify this transition of DWS probes and link it to our experimental measurements, we develop an extended decorated core-shell network (E-DCSN) model that couples the measured $\phi$-dependent self-motion plateau MSDs to the measured $1/\ell ^*(\phi )$, thereby enabling us to infer the effective average radius of the dominant scattering probes for different $\phi$. We then deduce the microrheological $G^\prime _\text {p}$ from solely optical measurements via the GSER. With these advances in interpreting DWS, passive microrheology delivers accurate $G^\prime _\text {p}$ for this highly complex, intermediately attractive dense emulsion system, as evidenced by comparisons with macroscopic rheometry, noting that the zero-frequency shear rigidity of the system effectively vanishes at $\phi$ corresponding to the minimum in the notch of $1/\ell ^*(\phi )$. In addition, we predict trends in the probability distributions of local coordination number over a wide range of $\phi$ for IA emulsions through a principal-component fit to $1/\ell ^*(\phi )$ based on the E-DCSN model.

## Results and analysis

### Diffusing wave spectroscopy

**Figure 1:**
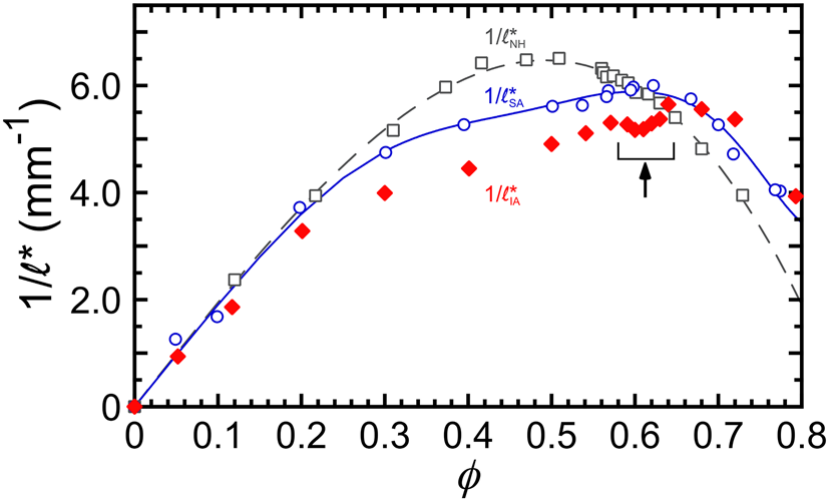
Measured inverse mean free path of optical transport, $1/\ell ^*$, of fractionated silicone oil-in-water (O/W) emulsions as a function of droplet volume fraction, $\phi$, for three different strengths of micellar depletion attraction (pathlength *L* = 5.0 mm, light wavelength $\lambda = 685$ nm). Intermediately attractive (this study, [SDS] = 35 mM, $|U_{\textrm{d}}| \approx$ 6 $k_{\textrm{B}}T$, average droplet radius $\langle a \rangle$ = 484 nm): $1/\ell ^*_\text {IA}$ (red solid diamonds). Nearly hard interactions (Kim et al.^47^, [SDS] = 10 mM, $|U_{\textrm{d}}| < k_{\textrm{B}}T$, similar $\langle a \rangle$ = 459 nm): $1/\ell ^*_\text {NH}$ (gray open squares); fit (gray dashed line) using Eq. (6) in Xu et al.^60^. Strongly attractive (Xu et al.^60^, [SDS] = 80 mM, $|U_{\textrm{d}}| \approx$ 15 $k_{\textrm{B}}T$, $\langle a \rangle$ = 459 nm): $1/\ell ^*_\text {SA}$ (blue open circles); fit (blue solid line) from Xu et al.^60^ using Eq. (5) in Xu et al.^60^ with constraints imposed by Eqs. (1-4) in Xu et al.^60^.

The measured $\phi$-dependent inverse mean free path of optical transport, $1/\ell ^*_\text {IA}$, for the intermediately attractive emulsions (Fig. 1, red diamonds) is asymmetric and has two knees, similar to $1/\ell ^*_\text {SA}$ for the strongly attractive emulsions (Fig. 1, blue circles). Strikingly, $1/\ell ^*_\text {IA}(\phi )$ also exhibits a notch-like dip (Fig. 1, bracket and arrow, 0.58 $\le \phi \le$ 0.64). By contrast, $1/\ell ^*_\text {NH}(\phi )$, measured for a very similar emulsion having nearly hard interactions at considerably lower [SDS] (Fig. 1, gray squares), has a much simpler inverted parabolic shape that peaks at $\phi \approx 0.50$. For the IA emulsion, in the low $\phi$ regime, 0 $< \phi \lesssim$ 0.20, the magnitude and slope of $1/\ell ^*_\text {IA}(\phi )$ are close to both $1/\ell ^*_\text {NH}(\phi )$ and $1/\ell ^*_\text {SA}(\phi )$. At larger $\phi$, a first knee in $1/\ell ^*_\text {IA}(\phi )$ is observed at $\phi \approx 0.2$, a slightly lower $\phi$-value than the first knee in $1/\ell ^*_\text {SA}(\phi )$. Above this first knee, $1/\ell ^*_\text {IA}(\phi )$ grows approximately linearly but with a lower slope than in the dilute $\phi$-regime; moreover, $1/\ell ^*_\text {IA}$ has a smaller magnitude than both $1/\ell ^*_\text {NH}$ and $1/\ell ^*_\text {SA}$, indicating that dense microscale emulsions with intermediate attractions over 0.2 $\lesssim \phi \lesssim$ 0.64 scatter less than emulsions having nearly-hard interactions and also strong attractions. At the upper end of this $\phi$-range, a dip-like notch is observed, and this notch region ends just before the second knee where the slope in $1/\ell ^*_\text {IA}(\phi )$ becomes negative towards the highest $\phi$ shown. Within the notch, a local minimum is observed at $\phi \approx$ 0.60. In the high-$\phi$ regime above the second knee, the magnitude of $1/\ell ^*_\text {IA}$ and $1/\ell ^*_\text {SA}$ are about the same, within experimental uncertainties in $\phi$, and both are greater than $1/\ell ^*_\text {NH}$. This implies that IA and SA emulsions scatter more in the strongly compressed high-$\phi$ limit than similar emulsions having NH interactions.

The optical transport results for the IA emulsion used in this present study can be directly compared with prior studies of nearly identical emulsions at other [SDS]^47,59,60^ because we have prepared a fractionated IA emulsion using the same materials and methods. The average hydrodynamic radius of the IA emulsion, $\langle a \rangle$ = 484 nm, is within 5% of $\langle a \rangle$ = 459 nm in the prior studies. Moreover, considering the Mie-scattering from an ideal isolated sphere, $1/\ell ^*_\text {ISA,Mie}$, given by independent scattering approximation (ISA) in the highly dilute limit, the difference between the IA emulsion and the prior studies is less than 0.5%. Therefore, the substantial differences in $1/\ell ^*(\phi )$, found between the present and prior studies as shown in Fig. 1, arise from different droplet structures caused by the different attractive strengths $|U_\text {d}|$, not from the very small difference in $\langle a \rangle$.

**Figure 2:**
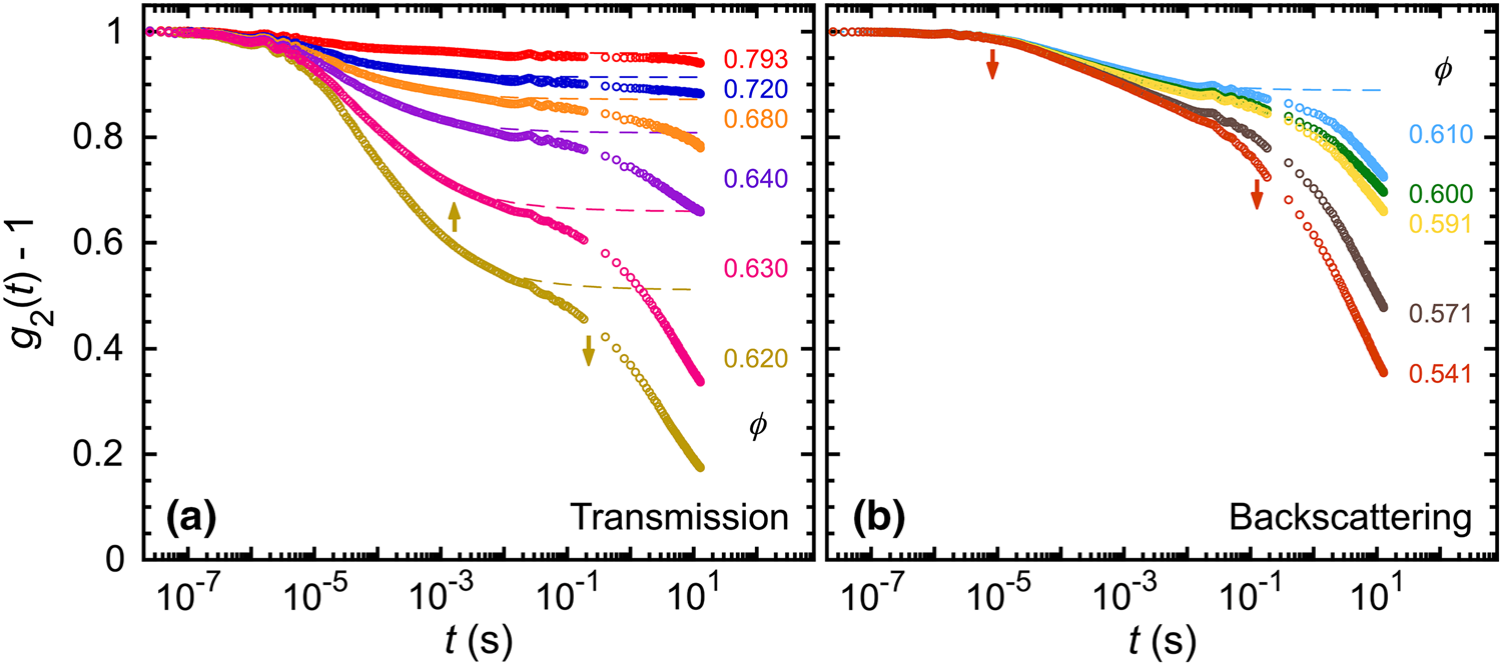
Measured time-dependent DWS intensity autocorrelation functions, $g_{\textrm{2}}(t) - 1$, of intermediately attractive O/W emulsions for dense droplet volume fractions $\phi$ (color-coded, see right), measured in (**a**) transmission and (**b**) backscattering geometries, respectively (pathlength *L* = 5 mm, light wavelength $\lambda$ = 685 nm). DWS multi-tau correlation data extend from early times up to about $2 \times 10^{-1}$ s; DWS echo data are shown at longer times. Arrows indicate the sign of concavity in the linear-log plot (up = +, down = −): [part (a)] for $\phi$ = 0.620, $g_{\textrm{2}}(t) - 1$ is concave up at earlier times, then concave down at longer times; [part (b)] by contrast, at lower $\phi$ = 0.541, no concave up region is observed (both arrows are down). Dashed lines correspond to the calculated primary decay-to-plateau $g_{\textrm{2}}(t) - 1$ using the fitting parameters obtained from the fits in Fig. 3a. Minor damped oscillatory noise signals, resulting from minute mechanical vibrations, are superimposed on the main $g_{\textrm{2}}(t) - 1$ signals, and visible for all $\phi$ at $3 \times 10^{-7}$ s $\lesssim t \lesssim 10^{-5}$ s and $5 \times 10^{-3}$ s $\lesssim t \lesssim 2 \times 10^{-1}$ s.

Each of the measured DWS intensity autocorrelation functions, $g_2(t)-1$, exhibits a primary decay at $t \approx 10^\text {-5}$ s, a primary plateau, and then a secondary decay at $10^\text {-1}$ s, considering both transmission and backscattering geometries, for the IA emulsion over a wide range of dense $\phi$ (Fig. 2). From prior DWS experiments by Kim et al.^59^ on dense MA emulsions at somewhat lower [SDS] = 20 mM, it has been hypothesized that secondary decays in DWS correlation functions can be attributed to a minor sub-population of droplets which are only marginally bound; this secondary decay gives rise to excess DWS MSDs at long times. While the details of the secondary decays are different for IA than MA emulsions, secondary decays observed in the present IA study also indicate the existence of excess MSDs. As with the MA emulsion, here for the IA emulsion, we seek to obtain primary plateau MSD values that can be gleaned from the measured DWS $g_2(t)-1$ at intermediate times prior to the secondary decays. We determine a plateau in the measured $g_2(t)-1$ by locating the transition between concave up and concave down features as shown in the linear-log format. This transition occurs at $t \approx 2 \times 10^\text {-2}$ s, as can be seen most easily at $\phi =$ 0.62 in transmission (Fig. 2a). The secondary decay in $g_2(t) - 1$ at longer time *t* $\gtrsim 2 \times 10^\text {-2}$ s can be attributed to the excess MSDs, which are not indicative of the average probe motion for microrheological purposes^59^. For $\phi \le$ 0.60, we cannot discern a transition from concave up to concave down; so, we refrain from identifying a plateau DWS MSD.

**Figure 3:**
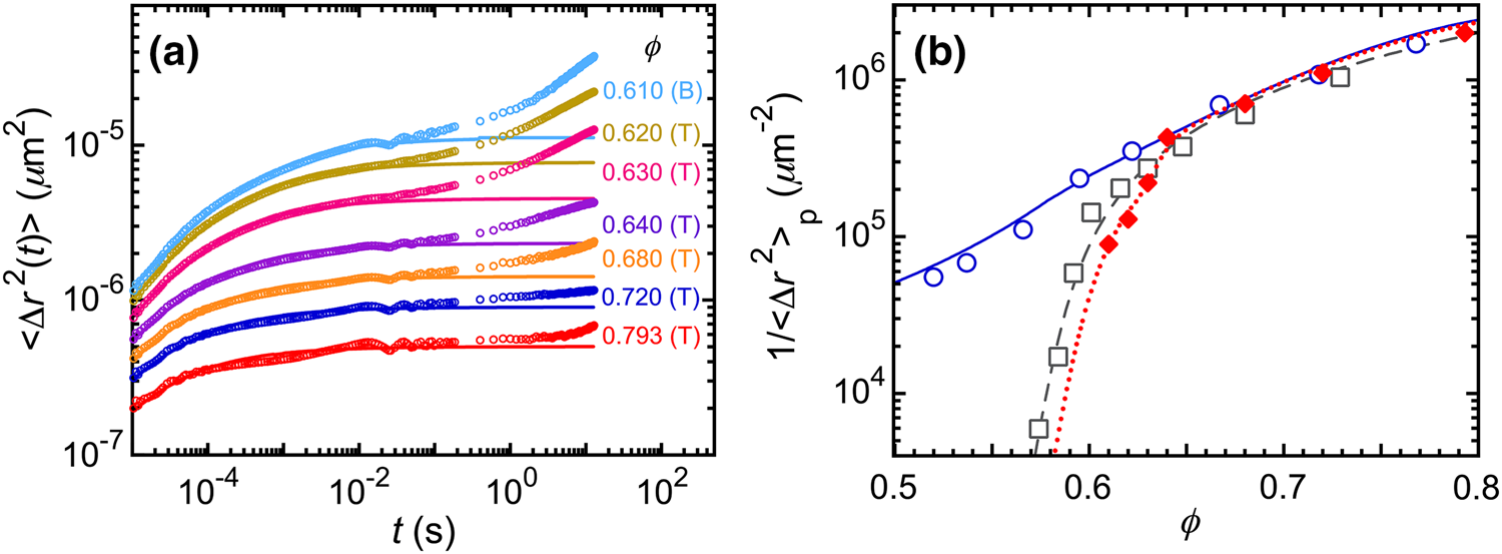
Time-dependent DWS mean square displacements (MSDs), $\langle \Delta {{\varvec{r}}}^2 (t) \rangle$, and inverse primary plateau MSDs, 1/$\langle \Delta {{\varvec{r}}}^2 \rangle _{\textrm{p}}$, as a function of $\phi$. (**a**) Ensemble-averaged $\langle \Delta {{\varvec{r}}}^2 (t) \rangle$ for a series of different $\phi$, extracted from $g_2(t)-1$ in Fig. 2 and corrected for collective scattering using $1/\ell ^*_\text {IA}$ in Fig. 1. Rises in the MSDs at long times are attributed to excess MSDs^59^, which do not reflect average probe dynamics. DWS MSDs at early-to-intermediate times are fit to an emulsion model, developed for nearly hard droplet interactions by Kim et al.^47^. Fits (solid lines) provide plateau self-motion probe MSDs, $\langle \Delta {{\varvec{r}}}^2 \rangle _{\textrm{p}}$. (T) and (B) indicate transmission and backscattering geometries, respectively. (**b**) 1/$\langle \Delta {{\varvec{r}}}^2 (\phi ) \rangle _{\textrm{p}}$ for emulsion systems having different types of interactions between droplets. Intermediate attractions (this study): 1/$\langle \Delta {{\varvec{r}}}^2 \rangle _{\textrm{p,IA}}$ (red diamonds), obtained from long-time plateau values of the fits in part (a). Dotted line: prediction curve with a smooth transition between an analytical interpolation of measured 1/$\langle \Delta {{\varvec{r}}}^2 \rangle _{\textrm{p,IA}}$ for $\phi \ge$ 0.61, and the rescaled calculation using the EEI model^27^ and GSER for $\phi<$ 0.61. Nearly hard interactions (Kim et al.^47^): 1/$\langle \Delta {{\varvec{r}}}^2 \rangle _{\textrm{p,NH}}$ (gray squares, dashed line: fit using the EEI model and GSER). Strong attractions (Xu et al.^60^): 1/$\langle \Delta {{\varvec{r}}}^2 \rangle _{\textrm{p,SA}}$ (blue circles, solid line: analytical interpolation).

To obtain the primary plateau MSDs, $\langle \Delta {{\varvec{r}}}^2\rangle _\text {p}$, for later use in the GSER of passive microrheology, we have extracted the probe MSDs $\langle \Delta {{\varvec{r}}}^2(t)\rangle$ from measured $g_2(t) - 1$; these MSDs are corrected for collective scattering using $1/\ell ^*_\text {IA}$ and the independent scattering approximation^47^. Each of the probe MSDs exhibits an early-time rise up to $t \approx 3 \times 10^\text {-5}$ s, a gradual bending to primary plateau, and then a secondary rise beyond $t \approx 2 \times 10^\text {-2}$ s (Fig. 3a). The primary plateau MSD values are obtained by utilizing the dense-emulsion MSD model^47^ to fit the early-time rise-to-plateau of $\langle \Delta {{\varvec{r}}}^2(t)\rangle$ for $\phi \ge$ 0.61, while compensating slightly for the periodic vibrational noise (*i.e.* damped oscillatory signal superimposed on what would otherwise be a smooth MSD) that is mostly pronounced at $10^\text {-2}$ s $\le t \le 10^\text {-1}$ s. The early-time MSD fitting curves are displayed as solid lines in Fig. 3a, and the corresponding calculated primary decay-to-plateau $g_2(t)-1$ are represented by dashed lines in Fig. 2.

We present the inverse primary-plateau probe MSDs , 1/$\langle \Delta {{\varvec{r}}}^2 \rangle _{\textrm{p}}$, which are proportional to the shear elastic plateau moduli in the GSER, as a function of $\phi$ for the NH^47^, IA, and SA^60^ emulsion systems in Fig. 3b. All of these emulsion systems have very similar droplet radii and polydispersities; only the interactions between droplets are different. After correcting for collective scattering, 1/$\langle \Delta {{\varvec{r}}}^2 \rangle _{\textrm{p}}$ of all the NH, IA, and SA systems, within the experimental uncertainties, are the same at higher $\phi \gtrsim$ 0.64 (Fig. 3b). Interestingly, as $\phi$ decreasing from 0.64, the 1/$\langle \Delta {{\varvec{r}}}^2 \rangle _\text {p}$ versus $\phi$ of these systems behave very differently. For the NH system, 1/$\langle \Delta {{\varvec{r}}}^2 (\phi ) \rangle _\text {p,NH}$ follows the prediction of the entropic, electrostatic, and interfacial (EEI) model^27^ with a knee on the log-linear plot located near $\phi \approx$ 0.60. Strikingly, 1/$\langle \Delta {{\varvec{r}}}^2 (\phi ) \rangle _\text {p,SA}$ is substantially larger than inverse plateau MSDs of both the NH and IA systems over 0.52 $\le \phi \lesssim$ 0.64, indicating highly restricted droplet motion in the SA system’s gel network of droplets even for $\phi$ well below $\phi _{\textrm{MRJ}}$. By contrast, 1/$\langle \Delta {{\varvec{r}}}^2 (\phi ) \rangle _\text {p,IA}$ is somewhat smaller than 1/$\langle \Delta {{\varvec{r}}}^2 (\phi ) \rangle _\text {p,NH}$ when $\phi \lesssim \phi _{\textrm{MRJ}}$, and it exhibits a rapid drop just below $\phi _{\textrm{MRJ}}$ as $\phi$ decreasing to 0.61. For $\phi<$ 0.61, the signal from excess MSDs is so strong that it interferes with our protocol to obtain primary plateau MSD values. Comparing to the NH system, the higher [SDS] in the IA system not only induces an intermediate strength of depletion attraction, but also leads to a smaller screening length that shifts the effective jamming point up in $\phi$, resulting in a smaller 1/$\langle \Delta {{\varvec{r}}}^2 (\phi ) \rangle _\text {p}$. The concave-up appearance of 1/$\langle \Delta {{\varvec{r}}}^2 (\phi ) \rangle _\text {p,IA}$ in log-linear format is similar to the shape of the upper end of the notch in $1/\ell ^*_\text {IA}(\phi )$ over the same $\phi$-range.

### Model

We hypothesize that an extended decorated core-shell network (E-DCSN) model, which takes into account scattering contributions from droplets having different local coordination numbers, *N*^16^, in different local regions, can describe the complicated shape of $1/\ell ^*_\text {IA}(\phi )$ for the disordered IA emulsion. Its predecessor, the DCSN model, had been introduced to describe and interpret the less complex shape of $1/\ell ^*_\text {SA}(\phi )$ of SA emulsions using only three principal components^60^. The lower attractive strength of $|U_\text {d}| \approx$ 5.6 $k_\text {B}T$ in the IA emulsion, as compared to $\approx$ 15 $k_\text {B}T$ in the SA emulsion, leads to a greater population of compact dense clusters and less tenuous gel structures in the IA emulsion. Moreover, the notch-like feature in $1/\ell ^*_\text {IA}(\phi )$ cannot be captured using only three principal components. While retaining the three principal components of the DCSN model, the E-DCSN model introduces a fourth principal component (*i.e.* non-percolating core clusters having high $\langle N \rangle$), which enables the notch-like dip in $1/\ell ^*_\text {IA}(\phi )$ to be captured. By interpreting the principal components of the E-DCSN model, we are able to obtain further insight into the dynamical optical fluctuations and passive microrheological interpretation of the IA emulsion. The four principal components in the E-DCSN model are: percolating core droplets, non-percolating core droplets, shell droplets, and surface decorating droplets (SDDs). We assume that only the percolating core droplets, which have high $\langle N \rangle \approx 12$, form a gel-like network, which can support macroscopic shear stresses elastically. In addition, there are non-percolating core droplets in the form of dense clusters, which have somewhat lower $\langle N \rangle \approx 9$ and do not participate in supporting macroscopic shear stresses elastically. Both types of core regions are typically surrounded by shell droplets, which have significantly smaller $\langle N \rangle \approx 6$. In turn, the shell regions are decorated with SDDs, which have $\langle N \rangle \approx 3$.

The scattering of the IA emulsion can be tied to the E-DSCN model through the relative scattering contributions of the four different principal components, each of which vary with $\phi$. Droplets having lower $\langle N \rangle$ effectively scatter more than droplets having higher $\langle N \rangle$ because less crowding leads to a larger scattering cross section. We parameterize the principal components using component droplet volume fractions of the different species: $\phi _\text {SDD}$ for SDDs, $\phi _\text {shell}$ for shell droplets, $\phi _\text {core,nonperc}$ for non-percolating core droplets, and $\phi _\text {core,perc}$ for percolating core droplets. As $\phi$ is varied, each of these principal component droplet volume fractions can change, reflecting structural changes in the IA emulsion from the dilute regime to the concentrated regime. To ensure droplet volume conservation, the total droplet volume fraction is the sum of the four $\phi$-dependent component droplet volume fractions: $\phi = \phi _\text {SDD}(\phi ) + \phi _\text {shell}(\phi ) + \phi _\text {core,nonperc}(\phi ) + \phi _\text {core,perc}(\phi )$. At a given $\phi$, we assume that scattering contributions from each of the four principal components are linear in their respective volume fractions in $1/\ell ^*_\text {IA}(\phi )$:

1 $$\begin{aligned} \begin{aligned} 1/\ell ^*_\text {IA}&= 1/\ell ^*_\text {SDD} + 1/\ell ^*_\text {shell} + 1/\ell ^*_\text {core,nonperc} + 1/\ell ^*_\text {core,perc} \\&= (1/\ell ^*_\text {SDD,0})(\phi _\text {SDD} + r_\text {shell}\phi _\text {shell} + r_\text {core,nonperc}\phi _\text {core,nonperc} +r_\text {core,perc}\phi _\text {core,perc}), \end{aligned} \end{aligned}$$

where $1/\ell ^*_\text {SDD,0}$ is the slope of linear growth in scattering at dilute $\phi$ where SDDs dominate; $r_\text {shell}$, $r_\text {core,nonperc}$ and $r_\text {core,perc}$ are the dimensionless relative scattering intensity from a shell droplet, from a non-percolating core droplet, and from a percolating core droplet, respectively, compared to a SDD.

**Figure 4:**
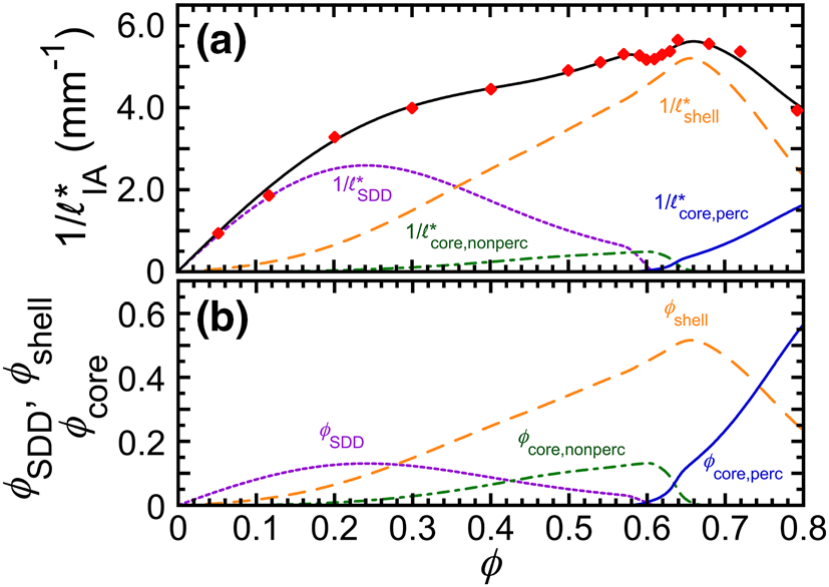
Principal component analysis of $1/\ell ^*_\text {IA}(\phi )$ using the extended decorated core-shell network (E-DCSN) model (see text). (**a**) Measured $1/\ell ^*_\text {IA}(\phi )$ (red diamonds) from Fig. 1. Fit: Eq. (1) (black solid line). Inferred $\phi$-dependent scattering contributions from: surface decorating droplets ($1/\ell ^*_\text {SDD}$, purple dotted line), shell droplets ($1/\ell ^*_\text {shell}$, orange dashed line), non-percolating core droplets ($1/\ell ^*_\text {core,nonperc}$, green dashed-dotted line), and percolating core droplets ($1/\ell ^*_\text {core,perc}$, blue solid line). (**b**) Component droplet volume fractions: $\phi _\text {SDD}$, $\phi _\text {shell}$, $\phi _\text {core,nonperc}$, and $\phi _\text {core,perc}$ [line colors and types as in part (a)].

Ignoring the notch-like dip, the overall shape of $1/\ell ^*_\text {IA}(\phi )$ is determined initially without introducing the non-percolating core droplets in a manner similar to the DCSN model^60^. We assume a linear rise in $\phi _\text {SDD}$ as $\phi$ increasing from 0 to $\approx$0.2, followed by an exponential decrease toward $\phi \approx$ 0.6. The initial rise in $\phi _\text {SDD}(\phi )$ originates from the formation of small clusters through slippery diffusion-limited cluster aggregation^15,16^, whereas the later decrease in $\phi _\text {SDD}(\phi )$ is attributed to the conversion of SDDs primarily to shell droplets. These features can be described using the formula: $\phi _\text {SDD}(\phi ) = \phi / \{1+\text {exp}[(\phi - \phi _\text {SDD,F})/\Delta \phi _\text {SDD}]\}$. As for $\phi _\text {core}(\phi )$ in the DCSN model of SA emulsions, here for IA emulsions, we deduce $\phi _\text {core,perc}(\phi )$ from an interpolation of the measured DWS inverse plateau MSDs, 1/$\langle \Delta {{\varvec{r}}}^2(\phi )\rangle _\text {p}$ (see the red dotted line in Fig. 3b). Again, based on the effective medium assumption inherent in the DCSN model, we assume that $\phi _\text {core,perc}$ is proportional to 1/$\langle \Delta {{\varvec{r}}}^2\rangle _\text {p}$ at a given $\phi$, and we set the effective probe radius to be 2$\langle a \rangle$ in the compressed regime at highest $\phi$ beyond the notch, where this is known to be the case when the DCSN model is applied to SA emulsions. Volume conservation of droplets, here continuing to exclude consideration of non-percolating core droplets, implies that $\phi _\text {shell}(\phi )$ can be determined from $\phi _\text {shell}(\phi ) = \phi - [\phi _\text {SDD}(\phi ) + \phi _\text {core,perc}(\phi )]$.

Having determined reasonable initial starting points for three of the four principal components, which broadly describe the overall shape of $1/\ell ^*_\text {IA}(\phi )$ without accounting for the notch, we then turn to the more complex aspect of modeling the notch of the IA emulsion. In particular, the existence of the notch can be interpreted as a consequence of a non-negligible population of non-percolating core droplets that lack adequate connectivity between constituent clusters, not the highly dense, well-interconnected cores of percolating gel-like networks of droplets. In effect, non-percolating core droplets reduce the scattering from the emulsion without contributing to its shear elasticity. We hypothesize that jamming of non-percolating dense clusters as $\phi$ is increased in the notch region leads to the creation of elastic shear-stress supporting networks that are less tenuous for the IA emulsion than for the SA emulsion. This cluster-jamming also is accompanied by a sharp reduction in SDDs towards the lower end of the notch, as clusters become closely proximate and SDDs are converted into shell and core droplets. Also, to account for the reduced magnitude of $1/\ell ^*_\text {IA}(\phi )$ over the wide range $\phi \le$ 0.64 as compared to the SA system in addition to the notch, we introduce the fourth component $\phi _\text {core,nonperc}$, which we assume quadratically increases from very low $\phi$, so as not to influence the low-$\phi$ slope in the scattering, and then exponentially decreases beyond $\phi \gtrsim$ 0.6. Weights are transferred to non-percolating droplets from SDDs and shell droplets, while preserving $\phi$ conservation of all four components. This procedure results in a good match to the measured $1/\ell ^*_\text {IA}(\phi )$ and also provides plausible, smooth $\phi$-dependences of the four principal components, as shown in Fig. 4a, recognizing that the solution to this problem is formally ill-posed given the available measurements. Although more principal components could be considered, even to the point of having separate components for each possible integer value of $\langle N \rangle$, the E-DCSN model with only four principal components describes all the key features of $1/\ell ^*_\text {IA}$ over all $\phi$, including the notch, through the smooth conversion between the component volume fractions of different species (Fig. 4). Given prior constraints on the relative scattering of different components, known from modeling SA emulsions, we set the optical transport parameters in Eq. (1) for this IA emulsion to be the same as was found for the SA emulsion: $1/\ell ^*_\text {SDD,0}$ = 19.8 mm$^{-1}$ and $r_\text {shell}$ = 0.509. Starting from $r_\text {core}$ = 0.150 for the SA system, we determine $r_\text {core, nonperc}$ = 0.187 and $r_\text {core, perc}$ = 0.145 for the IA system by taking into account the relative surface-to-volume ratio of the non-percolating and percolating core droplets and minimizing $\chi ^2$ for the least-squares fit of $1/\ell ^*_\text {IA}(\phi )$. The optimized parameters for $\phi _\text {SDD}(\phi )$ are determined to be $\phi _\text {SDD,F}$ = 0.265 and $\Delta \phi _\text {SDD}$ = 0.105 from the least-squares fitting of $1/\ell ^*_\text {IA}(\phi )$ at dilute $\phi$ where SDDs dominate. The detailed iterative procedure for obtaining smooth functions of the four principal components, while minimizing $\chi ^2$ for the least-squares fit of $1/\ell ^*_\text {IA}(\phi )$, is explained in the Methods and Supplementary Information.

**Figure 5:**
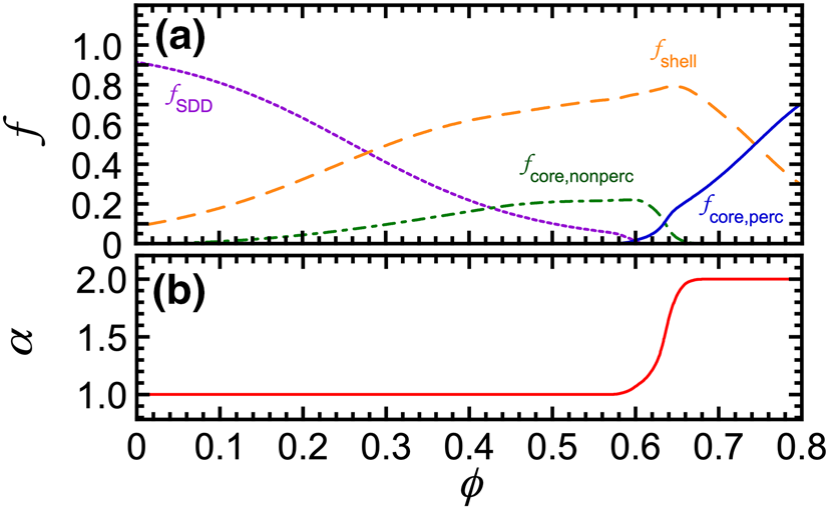
Droplet volume-fraction dependence of: (**a**) component relative volume-fractions, *f*, determined from Fig. 4a using the E-DCSN model (line types and colors: same as Fig. 4a), and (**b**) dimensionless effective DWS probe-size factor (see text), $\alpha$, obtained from core components in part (a), which ranges from 1 (single-droplet probe) to 2 (LDC probe).

As a different way of parameterizing the relative proportions of the four principal components, we also calculate the component relative volume fractions: $f_\text {SDD} = \phi _\text {SDD}/\phi$, $f_\text {shell} = \phi _\text {shell}/\phi$, $f_\text {core,nonperc} = \phi _\text {core,nonperc}/\phi$, and $f_\text {core,perc} = \phi _\text {core,perc}/\phi$ (Fig. 5a). We hypothesize that the component relative volume fractions of non-percolating and percolating core droplets can be used to determine the dimensionless $\phi$-dependent effective DWS probe-size factor via: $\alpha (\phi ) = \langle a_{\textrm{pr}} (\phi ) \rangle /\langle a \rangle = (f_\text {core,nonperc} + 2 f_\text {core,perc})/(f_\text {core,nonperc} + f_\text {core,perc})$, where $\langle a_{\textrm{pr}} (\phi ) \rangle$ is the $\phi$-dependent effective average radius of the DWS scattering probes. The resulting $\alpha (\phi )$, ranging from 2.0 in the high-$\phi$ limit to 1.0 in the low-$\phi$ limit, is shown in Fig. 5b. This hypothesis implies that the DWS scattering probes are effectively individual droplets at low $\phi$, similar to the MA emulsions at lower depletion strength than the IA emulsions, and it implies that the DWS scattering probes are effectively LDCs at high $\phi$ well above jamming, similar to the SA emulsions at higher depletion strengths. In the notch region, the relative proportions of these change rapidly, and $\alpha$ effectively represents an average over populations of single droplets and LDCs.

**Figure 6:**
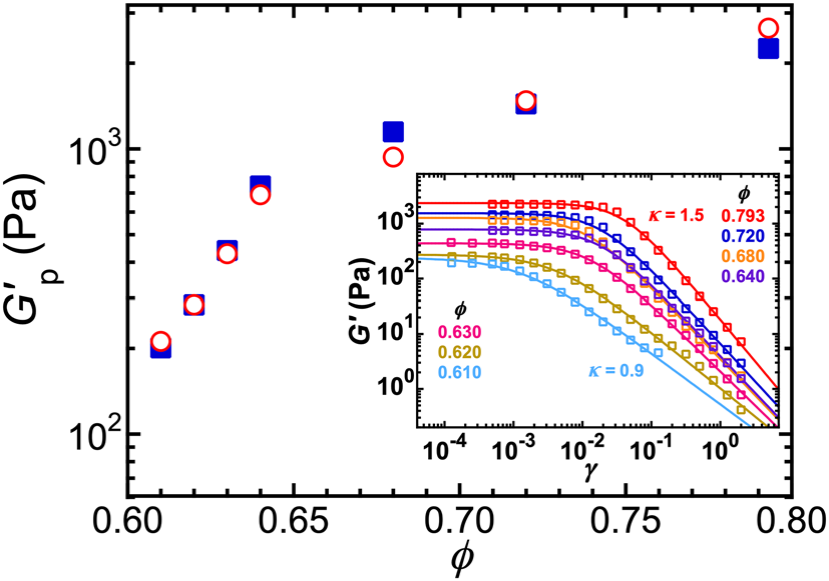
Comparison of DWS-GSER microrheological plateau elastic shear moduli, $G^\prime _\text {p,GSER}(\phi )$ (red open circles), obtained using plateau MSDs from Fig. 3a and the E-DCSN model’s $\alpha (\phi )$ from Fig. 5b, with plateau elastic shear moduli $G^\prime _\text {p,mech}(\phi )$ measured by mechanical rheometry (blue solid squares, from fits to strain-dependence in the inset). Inset: Mechanical shear oscillatory measurements of the storage modulus $G^\prime$ as a function of the applied peak strain amplitude $\gamma$ for intermediately attractive dense emulsions at [SDS] = 35 mM and frequency $\omega =1$ rad/s. Solid lines: fits using $G^\prime (\gamma )=G^\prime _\text {p,mech}/\left[ \left( \gamma /\gamma _\text {y}\right) ^\kappa + 1 \right]$, yielding the plateau shear modulus $G^\prime _\text {p,mech}$; yield strain $\gamma _\text {y}$ indicates the position of the knee that defines yielding; and high-strain power law parameter $\kappa$ describes the decrease in the non-linear $G^\prime$ well beyond yielding.

### Comparison of DWS microrheology with mechanical macrorheology

Based on the above hypothesis regarding the DWS probe-size factor, $\alpha$, which is tied to the E-DCSN analysis of the measured optical transport properties, we use the GSER to calculate the low-frequency plateau elastic shear moduli, $G^\prime _\text {p,GSER}(\phi )$, of the IA emulsion:

2 $$\begin{aligned} {} G^\prime _\text {p,GSER}(\phi ) = \frac{k_\text {B}T}{\pi \langle a_{\textrm{pr}} (\phi ) \rangle \langle \Delta {{\varvec{r}}}^2 (\phi ) \rangle _\text {p}} = \frac{k_\text {B}T}{\pi [\alpha (\phi )] \langle a \rangle \langle \Delta {{\varvec{r}}}^2 (\phi ) \rangle _\text {p}}. \end{aligned}$$

We compare this result with mechanical rheometry measurements in Fig. 6. We find good quantitative agreement between the mechanical $G^\prime _\text {p,mech}$ and microrheological $G^\prime _\text {p,GSER}$ over all $\phi$ probed. Use of $\phi$-independent $\alpha$ of either 1 or 2 leads to small but systematic deviations that are apparent at either high or low $\phi$, respectively. Of all emulsions that we have studied systematically thus far (*i.e.* NH, MA, IA, and SA), the IA emulsion is the most challenging to interpret, both in regards to optical transport properties and also to passive microrheology.

### Probability distribution of local coordination number

At each $\phi$, we infer discrete probability distributions, $p_N(N)$, using the results of the principal component analysis in the E-DCSN model. For each principal component (*i.e.* SDDs, shell droplets, non-percolating core droplets, and percolating core droplets), we assume Gaussian distributions as a function of *N* centered on $\langle N \rangle$ = 3, 6, 9, and 12, respectively, each with a standard deviation $\sigma _N$ = 2.5. The corresponding probability density functions are denoted as $p_\text {SDD}(N)$, $p_\text {shell}(N)$, $p_\text {core,nonperc}(N)$, and $p_\text {core,perc}(N)$. Here, $p_N(N \le 2)$ has been folded toward larger *N*, corresponding to the known distribution of *N* for slippery diffusion-limited cluster aggregation (S-DLCA)^16^. At each $\phi$, $p_N(N)$ is the sum of these probability density functions, each of which is weighted by its corresponding component volume fraction and normalized by the total $\phi$:

3 $$\begin{aligned} p_N(N,\phi ) = [\phi _\text {SDD}(\phi )p_\text {SDD}(N) + \phi _\text {shell}(\phi )p_\text {shell}(N) + \phi _\text {core,nonperc}(\phi )p_\text {core,nonperc}(N) + \phi _\text {core,perc}(\phi )p_\text {core,perc}(N)]/ \phi . \end{aligned}$$

**Figure 7:**
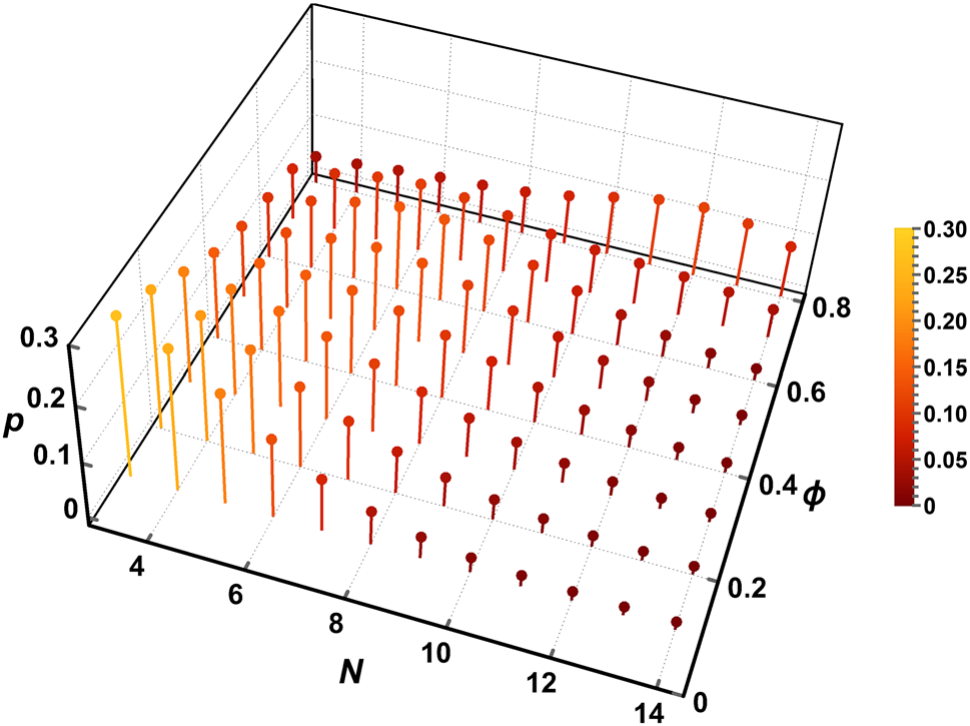
Normalized probability distributions of local coordination numbers, $p_N(N)$, for different volume fractions $\phi$, inferred from the components of the E-DCSN model in Fig. 4b (see text for details). For this intermediately attractive emulsion, $p_N(N)$ shifts from being low-*N* dominant at low $\phi$, to mid-*N* dominant at $\phi \approx 0.5$, and then to high-*N* dominant at high $\phi$

The results of these calculations are shown in Fig. 7. The chosen $\sigma _N$ = 2.5 yields smooth distributions at all $\phi$ shown; smaller $\sigma _N$ leads to distributions that have local maxima and are less smooth. Here, the definition of *N* corresponds most closely to an effective coordination number (*i.e.* number of slippery attractive bonds corresponding to near-contact between neighboring droplet interfaces), which is sometimes denoted *z* in other literature^45^. We emphasize that interdroplet bonding and near-contact configurations inherent in this definition of *N* are those that are most influential in determining optical transport properties (*i.e.* scattering) as well as in determining mechanical shear rigidity. Also, the substantial values in these distributions for $N>$ 12 towards high $\phi$ are facilitated by droplet deformation, arising from at least one of short-range attraction and osmotic compression; yet, regions of near-contact are stabilized against coalescence by the screened electrostatic repulsion. The presented $p_N(N,\phi )$ are consistent with our particular experimental protocol for emulsion-preparation and observed optical transport properties; yet, these distributions are not necessarily universal for any method of preparing an attractive emulsion.

## Discussion

Attractive colloidal systems are complex and rich, yet are also among the most challenging to explore experimentally. The precise structure of an attractive colloidal system can depend sensitively on the method of its preparation, and aging can sometimes occur and lead to slow structural evolution. Differences in preparation of short-range attractive colloidal systems can lead to non-unique structures and physical properties, even for monodisperse colloids at the same $\phi$ and same $|U_\text {d}|$. Here, by developing and systematically applying a protocol for preparing dense attractive emulsion systems that have slippery short-range attractions, we have been able to reveal important new features in the optical transport properties and DWS MSDs that accompany intermediate attractions. Our results for IA emulsions differ substantially from either MA or SA emulsions; yet, some known aspects of MA and SA emulsions are useful in understanding IA emulsions: excess DWS MSDs at long time (MA emulsions), and a DWS probe-size factor of $\alpha \approx 2$ at high $\phi$ above $\phi _\text {MRJ}$ (SA emulsions). However, the $\phi$-dependent optical scattering of the IA emulsion has a very interesting and striking notch near the random jamming point that has not be reported for either MA or IA emulsions. This notch, as interpreted using the E-DSCN model, suggests that a cluster-jamming scenario likely occurs as $\phi$ is raised, rather than either a droplet-jamming scenario for NH emulsions or a gel-network forming scenario for SA emulsions. In addition, this notch provides an important clue that signals different proportions of local dense structures and different interconnectivity of those local dense structures for IA emulsions, as compared to both MA and SA emulsions. The overall lower scattering of the IA emulsion at $\phi$ below jamming indicates that loosely bound attractive clusters, which have a lower surface-to-volume ratio, exist in the IA emulsion, as compared to the more tenuous, gel-like network structures of the SA emulsion. Moreover, the measured low-frequency plateau shear rigidity of the IA emulsion does not extend for $\phi$ below the minimum in the notch, whereas at substantially stronger attractive strengths, such low-frequency shear rigidity of the SA emulsion does extend well below jamming. This is further confirmed microrheologically from the measured DWS MSDs of the IA emulsion, after accounting for the existence of long-time excess MSDs. Using emulsions, rather than solid particulates, has facilitated all of these advances in understanding, since emulsions can be concentrated and manipulated at very high $\phi$ without the problems with irreversible aggregation that can be present in solid particulate systems.

Our results for the IA emulsion imply a complex and non-monotonic behavior of the $\phi$-dependent optical transport properties of concentrated emulsions as a function of $|U_\text {d}|$. Very low strengths of depletion attractions relative to thermal energy in MA emulsions do not yield substantial changes in scattering compared to emulsions with nearly hard interactions. However, the limit of very high depletion strengths relative to thermal energy in SA emulsions does not yield the greatest possible reduction in scattering at intermediate $0.45 \le \phi \le 0.55$. Instead, among MA, IA, and SA emulsions, we find that the greatest reduction in scattering is actually present in this range of $\phi$ for IA emulsions. So, the full behavior of this non-monotonic trend deserves further consideration, including a more detailed set of measurements at other $|U_\text {d}|$.

For IA emulsions, a $\phi$-dependent DWS probe-size factor $\alpha$, which varies from 1 in the notch region to 2 above the notch region, yields the best comparison with mechanical measurements. This probe-size factor reflects the relative proportions of scatterers that lead to the DWS signals which are interpreted as probe MSDs; the relative populations of scatterers in the cluster-jamming scenario of IA emulsions vary rapidly in the cluster-jamming regime corresponding to the notch in the optical transport. This DWS probe-size factor has been inferred from the measured optical transport and dynamic correlation functions by connecting these measurements to shear-rigidity percolation, which is linked to droplet coordination number through the principal component analysis and physical interpretation of the E-DCSN model. A full theoretical treatment of the DWS probe-size factor as a function of $\phi$ and $|U_\text {d}|$ is needed in order to establish its fundamental origin as well as to further examine the hypothesis that it can be determined through a ratio related to the fraction of non-percolating and percolating core droplets.

Overall, our results point to a cluster-jamming scenario for the IA emulsion. At lower $\phi$, clusters of droplets in the IA emulsion are compact and not strongly bonded together; the weak association of these clusters precludes the existence of a low-frequency plateau shear rigidity for the IA emulsion. However, as $\phi$ is raised, these clusters are forced to jam together, yielding plateau shear-rigidity, yet with a lower surface-to-volume ratio of the network structures as compared to the more tenuous networks of SA emulsions. Thus, it would be interesting to perform measurements over a broader range of $|U_\text {d}|$ to see how the cluster-jamming scenario of the IA emulsion transitions into the gel-network scenario of the SA emulsion. Likewise, it would be useful to explore lower $|U_\text {d}|$ to determine where the notch-like feature in $1/\ell ^*(\phi )$ emerges.

As a consequence of our experimental protocol, dense IA emulsions have been formed by diluting the concentrated master emulsion sample and shear-agitating at constant $|U_\text {d}|$ to form a system that is uniform in $\phi$. The agitation does not rupture droplets but instead only alters the droplets’ positional structure. This process of dilution and shear agitation at constant micellar concentration initially causes unjamming of attractive clusters that were originally jammed by osmotic compression through ultracentrifugation in the concentrated master emulsion. Subsequently, during the waiting time that we have designed into this protocol, attractive bonding between clusters can occur and gel-network formation is possible. For the SA system, to which the same experimental protocol has been applied, shear-rigid gel-like elastic networks form after disruption at $\phi$ well below $\phi _\text {MRJ}$ in the presence of strong $|U_\text {d}|$ $\approx$ 15 $k_\text {B}T$. However, for the IA emulsion having $|U_\text {d}|$ $\approx$ 6 $k_\text {B}T$, the weak association of clusters after disruption does not yield shear-rigidity percolation at the intermediate strength of attraction for $\phi$ extending well below $\phi _\text {MRJ}$, as is evident in the measured $G^\prime _\text {p}(\phi )$ when compared to the SA emulsion. This interpretation is consistent with both macroscopic shear rheometry measurements as well as our passive microrheological DWS measurements.

Our results, analysis, and interpretation for IA emulsions point to many exciting future directions, both in simulations and experiments. For instance, simulations could be developed to treat slippery attractive colloidal systems formed by diluting osmotically compressed attractive emulsions, imposing shear disruption at constant $|U_\text {d}|$, allowing subsequent cluster association and gel-network formation, and then exploring optical and mechanical responses with and without shear deformation. Such simulations would more closely align with our experimental protocols and observations than just quenching-in a secondary attraction in a homogeneous dispersion. In addition, the influence of minor populations of more mobile droplets on DWS signals could be identified and studied as a function of $|U_\text {d}|$ and $\phi$. Experimentally, one could potentially extend a reported confocal microscopy study that utilizes both refractive index-matched and density-matched, size-fractionated emulsions^35^ by inducing a short-range attraction and varying $\phi$ at different fixed values of $|U_\text {d}|$. In addition, further exploration of optical transport properties for values of $|U_\text {d}|$ in between IA and MA and also in between IA and SA will reveal the range over which the notch feature in $1/\ell ^*(\phi )$ can be observed.

In summary, our study provides a comprehensive set of experimental measurements of optical transport, rheological, and dynamic correlation functions for an intermediately attractive, dense system of fractionated, uniform emulsion droplets. Our experimental protocols have been refined to give reproducible results that enable direct quantitative microrheological comparisons of plateau elastic shear moduli that are not dominated by aging effects. We have found that discounting excess MSDs as well as utilizing a $\phi$-dependent DWS probe-size factor are both needed to obtain the best possible match of the passive microrheological interpretation of all measurements, when compared to macroscopic shear rheological measurements. To explain the additional complexity in the measured optical scattering, it has been necessary to include a fourth principal component in the E-DCSN model. Taken together, both the data and the modeling point to highly complex IA system in which loosely connected dense clusters, not isolated droplets, jam as $\phi$ is raised. We anticipate that future work will reveal additional finer details in the transition between MA, IA, and SA regimes as $|U_\text {d}|$ is varied and will provide insight into the fundamental origin of the DWS probe-size factor in short-range attractive colloidal systems.

## Methods

### Size-fractionated attractive emulsions

By microfluidic homogenization, we prepare a uniform silicone oil-in-water (O/W) microscale emulsion (poly-dimethyl siloxane oil, Gelest Inc.; viscosity: $\nu _\text {o}= 350$ cSt), SDS (Fisher Scientific; electrophoresis grade 99% purity), and deionized water (Millipore Milli-Q Academic; resistivity: 18.2 M$\Omega$ cm), as follows (see also Supplementary Information). We size-fractionate an initial polydisperse emulsion four times and repeatedly centrifuge to obtain a concentrated master ‘stock’ emulsion at fixed bulk [SDS] = 35 mM in a manner similar to Kim et al.^47^. Dynamic and static light scattering measurements provide primary characteristics of the droplet radial size distribution: average hydrodynamic radius $\langle a \rangle = 484 \pm 12$ nm and polydispersity $\delta a/\langle a\rangle \simeq 0.15$, where $\delta a$ is the standard deviation of this distribution. Using a gravimetric evaporation method^63^, we determine that the oil droplet volume fraction of the master emulsion is $\phi _\text {m} = 0.793 \pm 0.003$. Emulsions at lower $\phi$ are obtained by diluting this master emulsion with 35 mM SDS solution and stirring until uniform (see Supplementary Information).

### Optical transport and diffusing wave spectroscopy measurements

We perform optical transport and DWS measurements (Rheolab 3 light scattering instrument equipped with backscattering option, wavelength $\lambda$ = 685 nm; LS Instruments, Fribourg CH) to obtain the optical transport mean free path $\ell ^*$ and the transmission and backscattering intensity correlation functions for IA emulsions at different $\phi$. Each emulsion is loaded into a glass optical cuvette having a pathlength of *L* = 5 mm; a $\phi$-dependent loading protocol is used to eliminate air bubbles while avoiding gradients in $\phi$ (see Supplementary Information). Each loaded cuvette is then placed in the Rheolab 3 and allowed to equilibrate at a set temperature $T = 20 \pm 0.1$ $^\circ$C. The waiting time is 24 hours for $\phi \ge$ 0.4 and 1,200 s for $\phi<$ 0.4 (see Supplementary Information). Time-averaged backscattering and transmission intensities are used to determine $\ell ^*$. For $\phi \ge$ 0.4, fluctuating intensities in either the transmission or the backscattering detectors are used to determine $g_2(t) - 1$. For each emulsion sample at a given $\phi$, a total of at least 8 trials, each containing 300 s of multi-tau duration and 60 s of echo duration, have been performed and averaged. We extract the apparent $\langle \Delta {{\varvec{r}}}^2_\text {a} (t)\rangle$ by solving the classic transcendental equation of DWS^47,58,64^, using the measured $g_2(t) - 1$ and $\ell ^*$. Then, at each $\phi$, we correct the apparent MSD for collective light scattering effects using the measured $\ell ^*$, yielding the DWS probe’s MSD, $\langle \Delta {{\varvec{r}}}^2 (t)\rangle$ (see Supplementary Information).

### Mechanical shear rheometry

We measure the plateau elastic shear moduli, $G^\prime _\text {p,mech}$, using a controlled-strain mechanical shear rheometer (RFS-II, Rheometric Scientific, 25 mm diameter stainless steel cone-and-plate geometry, equipped with a vapor trap). At a frequency $\omega = 1$ rad/s, we measure and fit strain sweeps to obtain $G^\prime _\text {p,mech}$ of the IA emulsion at each $\phi$ (see also Supplementary Information).

### Regularized fitting using the extended decorated core shell network model

Consistent with the protocol for preparing emulsion samples at each $\phi$ from a concentrated master emulsion, we hypothesize that percolating core structures have an average $\phi _\text {net,core}$ = 0.793, the same value as the measured $\phi _\text {m}$ of the concentrated master emulsion, and we calculate $G^\prime _\text {p,EEI}(\phi _\text {net,core})$ using the EEI model^27^. We then deduce the magnitude of $\phi _\text {core,perc}(\phi )$ based on an effective medium assumption: $\phi _\text {core,perc}(\phi ) = \phi _\text {net,core} [G^\prime _\text {p,GSER}(\phi ) / G^\prime _\text {p,EEI}(\phi _\text {net,core})]$, where we determine $G^\prime _\text {p,GSER}(\phi )$ from the measured DWS plateau MSD with the assumption of a DWS probe size factor $\alpha \approx 2$ (assuming that IA emulsions are similar to SA emulsions at high $\phi$) in the GSER: $G^\prime _\text {p,GSER}(\phi ) = k_\text {B}T / [\pi \alpha \langle a \rangle \langle \Delta {{\varvec{r}}}^2(\phi )\rangle _\text {p}]$. Before introducing the fourth non-percolating core droplet component, we minimize $\chi ^2$ of the nonlinear least-squares fit for $1/\ell ^*_\text {IA}(\phi )$, only considering the dilute limit $\phi < 0.3$ and the high $\phi$ regime beyond the notch $\phi$ > 0.64, by varying the model’s parameters [Eq. (1) with $\phi _\text {core,nonperc}$ temporarily set to zero]. Then, after fitting the overall shape of $1/\ell ^*_\text {IA}(\phi )$ excluding the notch, we add in the $\phi _\text {core,nonperc}$ component by transferring weights from $\phi _\text {shell}$ for $\phi \le$ 0.64 and from $\phi _\text {SDD}$ within the lower end of notch region. After iterations of minimizing $\chi ^2$ for the $1/\ell ^*_\text {IA}(\phi )$ fit over the entire $\phi$ range, having all key features considered, we obtain a regularized curve fit of $1/\ell ^*_\text {IA}(\phi )$ with smooth conversion between all the component droplet volume fractions (see also Supplementary Information).

## Supplementary Information

Supplementary Information.

## Data Availability

The authors confirm that the data supporting the findings of this study are available within the article.
