# Supplementary material for: Complex optical transport, dynamics, and rheology of intermediately attractive emulsions
Source: Sci Rep. 2023 Jan 31;13:1791. doi: 10.1038/s41598-023-28308-6 (PMC9889356; doi:10.1038/s41598-023-28308-6)
Supplement: Supplementary file 1 — Supplementary Information. [file 41598_2023_28308_MOESM1_ESM.pdf]

Supplementary Information
Complex optical transport, dynamics, and rheology of intermediately attractive emulsions

Y. Xu and T. G. Mason

Attractive emulsion preparation, handling, and characterization

We prepare a uniform, size-fractionated emulsion having a short-range intermediately attractive depletion interaction ($|U_\mathrm{d}| \approx 5.6\ k_\mathrm{B}T$) using trimethylsiloxy terminated poly-dimethylsiloxane oil (PDMS, Gelest Inc.; kinetic viscosity: $\nu_\mathrm{o} = 350$ cSt; mass density: $\rho_\mathrm{o} = 0.97$ g cm^{-3}; average molecular weight: MW$_\mathrm{o}$ = 13,650 g mol^{-1}; refractive index: $n_\mathrm{o} = 1.4031$), sodium dodecyl sulfate (SDS, Fisher Scientific; electrophoresis grade 99% purity), and deionized water (Millipore Milli-Q Academic; resistivity: 18.2 MΩ cm). We follow the protocol of emulsification, homogenization, and size-fractionation developed by Kim *et al.*[47], but here fix [SDS] = 35 mM and thereby obtain a 4× size-fractionated, concentrated master emulsion. A small portion of this master emulsion is diluted in a 10 mM aqueous SDS solution to $\phi \approx 10^{-4}$ and then characterized using dynamic and static light scattering, yielding an average radius $\langle a \rangle = 484 \pm 12$ nm and polydispersity $\delta a/\langle a \rangle \simeq 0.15$, where δa is the standard deviation of the droplet radial size distribution. The emulsion in the present study of intermediate attractions (IA) at [SDS] = 35 mM has been purposefully made to be as close to the same as possible when compared to the emulsion used in recent prior DWS studies of nearly hard (NH)[47] at [SDS] = 10 mM, moderate attractions (MA)[59] at [SDS] = 20 mM, and strong attractions (SA)[60] at [SDS] = 80 mM. These NH, MA and SA emulsions have been made from PDMS having the same molecular weight as we used for the IA emulsions; moreover, the NH, MA and SA emulsions all have the same average radius $\langle a \rangle = 459 \pm 15$ nm and polydispersity $\delta a/\langle a \rangle \simeq 0.18$. So, the measured characteristics of the size distributions are very close, confirming that the IA emulsion is nearly the same as the emulsion used in prior NH, MA, and SA studies. Based on results of a prior optical study[63], the refractive index of the continuous phase of the IA emulsion at [SDS] = 35 mM is calculated to be $n_\mathrm{c} = 1.3320$ at room temperature T = 20.0 °C.

Setting [SDS] = 35 mM in the continuous phase, well above SDS's critical micelle concentration of $C^* \approx 8.1$ mM, induces lubricated depletion attractions between droplets in the fractionated PDMS O/W emulsion, resulting in a secondary minimum in the pair interaction potential. By combining Asakura-Oosawa's theory for larger and smaller hard spheres with Vrij's linear micellar model[8], the magnitude of this potential minimum is calculated to be $|U_\mathrm{d}| \approx 5.6\ k_\mathrm{B}T$. In addition to providing micellar depletion attractive interactions, adsorbed dodecyl sulfate anion (DS$^-$) on the droplet surfaces, as well as DS$^-$ and Na$^+$ in the continuous phase, also lead to a strong short-range Debye-screened-charge repulsion between droplets, which precludes their coalescence. The interdroplet attraction is lubricated and slippery as a result of the retention of an aqueous continuous phase layer between droplets; this is dissimilar to bonding between solid colloids that have been destabilized in a way that allows them to fuse together in a shear-rigid fashion as a result of exceedingly strong van der Waals attractions. The Debye screening length at $T = 20.0$ °C is $\lambda_\mathrm{D} \approx 1.64$ nm at [SDS] = 35 mM in the IA emulsion, as compared to $\lambda_\mathrm{D} \approx 3.04$ nm at [SDS] = 10 mM for the NH emulsion and to $\lambda_\mathrm{D} \approx 1.08$ nm at [SDS] = 80 mM for the SA emulsion. These changes in Debye screening length influence the shape and location of the rapid rise in the stabilizing repulsion between droplets, at shorter range than that corresponding to the secondary minimum cause by depletion effects. So, changing [SDS] alters not just $|U_\mathrm{d}|$ but also λ_D, which plays an important role in repulsive droplet jamming at higher applied osmotic pressures.

We concentrate the emulsion by centrifugation using a swinging bucket rotor (Beckman L8-55 ultracentrifuge, SW-28 swinging bucket rotor, 10,000 rpm, 1.25 h) without inducing droplet coalescence. We then separate and combine the concentrated elastic plugs formed at the top of centrifuge tubes; next, we dilute these combined recovered plugs to $\phi \approx 0.1$ using 35 mM SDS solution. We repeat this process twice more to set the SDS concentration and acquire a large volume of concentrated stock emulsion that is elastic at high ϕ. To avoid any size separation that could be caused by centrifugation, this concentrated master emulsion is thoroughly mixed at sufficiently low strain rates to prevent droplet rupturing. To reduce water loss from the master sample, we store this master sample in a temperature-controlled chamber set at a temperature of 20.0 °C to minimize the possible evolution of ϕ due to evaporation-condensation of water vapor into the cap and walls of the container, as well as water vapor leakage when opening and re-sealing the container. The oil droplet volume fraction of this concentrated master emulsion is measured to be $\phi_\mathrm{m} = 0.793 \pm 0.003$, determined using a gravimetric evaporation method[63].

We make IA emulsions at lower ϕ by diluting this concentrated master emulsion with an aqueous 35 mM SDS solution using an analytical balance (Denver Instruments APX-200, 0.1 mg precision). We stir each diluted sample having $\phi < \phi_\mathrm{m}$ using a small spatula, imposing an estimated 50 s^{-1} average shear flow-rate, for 3 minutes in a 3 mL vial to ensure that the resulting emulsion is homogeneously dispersed before loading into an optical cuvette or the mechanical rheometer. After

mixing, each diluted sample is split into two portions: 1.5 mL for optical transport and DWS measurements and 0.5 mL for mechanical rheometry. This guarantees that exactly the same sample with identical ϕ has been used for both DWS and mechanical rheometry measurements, which is necessary in order to make accurate microrheological comparisons.

Optical transport and diffusing wave spectroscopy measurements

Optical transport and DWS measurements are performed with a Rheolab 3 light scattering instrument (LS Instruments, Fribourg CH), equipped with backscattering option. Laser light (wavelength λ = 685 nm), transmitted from a rotating ground-glass diffuser, is subsequently collimated to illuminate the sample. At a given ϕ, each emulsion is loaded into a clean glass optical cuvette with a width of 10 mm and a thickness (*i.e.* pathlength) of L = 5 mm. This pathlength is large enough to ensure that the ℓ^* is always at least a factor of 4 smaller than L for all ϕ corresponding to the MSDs that we report. To avoid artifacts that could occur because of an inadequate sample volume, we ensure that the upper surface of the loaded emulsion is at least 15 mm high relative to the bottom surface of the cuvette.

The protocols that we have developed for loading emulsion samples into the cuvette and for setting the waiting time, which starts at the completion of sample loading and ends at the beginning of measurements, depend on how viscous or elastic each sample is and therefore on ϕ. For dilute viscous samples having $\phi \leqslant 0.3$, we only measure and report optical transport ℓ^*, not DWS MSDs, using the following protocol that has been designed to not be excessively influenced by gravitational compaction leading to phase separation, which is known for dilute attractive emulsions[62]. After dilution and mixing, we pour the sample into the cuvette over one wall, cap and seal the cuvette with Parafilm, and allow the sample to rest for 24 hours at 20.0 °C. Then, the cuvette is gently inverted for 5 times and finally righted. After placing the cuvette into the Rheolab 3, we wait 1,200 s before measuring ℓ^*. This waiting time of 1,200 s is significantly larger than the longest doubling time $\approx$1.7 s for the lowest ϕ = 0.052, calculated on basis of colloidal diffusion of droplets in water. Therefore, local dense clusters of droplets have adequate time to form through S-DLCA[15,16] for all presented $0.052 \leqslant \phi \leqslant 0.30$. At each ϕ, the reported $1/\ell^*$ is averaged from 11 trials and the trial-to-trial standard deviation is less than 4%. No noticeable creaming, which would create a systematic trend in the measured ℓ^*, is observed as a function of trial number.

We measure both ℓ^* and DWS $g_2(t)-1$ for all samples having $\phi \geqslant 0.401$ after loading the sample in the following manner to avoid introducing air bubbles into the cuvette. For $0.401 \leqslant \phi \leqslant 0.541$, the yield stress of the emulsion is still low enough that we can simply pour the emulsion into the cuvette over one wall. For $0.571 \leqslant \phi \leqslant 0.630$, we use a syringe with a stainless-steel needle (inner diameter I.D. = 0.84 mm) to load the samples. We begin by completely inserting the syringe tip into the bottom of the cuvette, then slowly withdrawing the syringe while injecting the emulsion and ensuring that the tip stays below the emulsion's surface. For $\phi \geqslant 0.64$, we transfer the emulsion with a small spatula into the cuvette as close as possible to its bottom; then, we use low speed (< 1,500 rpm) centrifugation for a total duration less than 60 s to eliminate stray air bubbles in loaded cuvettes without generating gradients in ϕ. After loading, the capped optical cuvettes are sealed with Parafilm, and all of the emulsion samples are stored in a temperature-controlled chamber for 24 hours to equilibrate before measurements. The sample temperature in the Rheolab 3 is maintained at $T = 20.0 \pm 0.1$ °C for all measurements. For each $\phi \geqslant 0.401$, we measure ℓ^* followed by DWS $g_2(t)-1$ in transmission geometry for 11 trials; and then we measure DWS $g_2(t)-1$ in backscattering geometry for 11 trials. Each trial of $g_2(t)-1$ measurements contains 300 s of multi-tau duration and 60 s of echo duration. The reported $1/\ell^*$ and $g_2(t)-1$ at each ϕ are obtained by averaging. The standard deviations of $1/\ell^*$ for all ϕ explored over $\phi \geqslant 0.4$ and also the standard deviations of the long-time g_2-1 for all ϕ explored over $\phi \geqslant 0.61$ are less than 2% of the corresponding average values.

At each ϕ, using the measured $g_2(t)-1$ and ℓ^*, we extract the apparent $\langle \Delta \boldsymbol{r}_\mathrm{a}^2(t) \rangle$ by solving the classic transcendental equation of DWS[47,58,64], and then each apparent MSD is converted into the probe self-motion $\langle \Delta \boldsymbol{r}^2(t) \rangle$ to correct for collective light scattering. The apparent MSD is multiplied by a dimensionless ratio, less than unity, given by the actual measured scattering strength at that ϕ, reflected by $1/\ell^*(\phi)$, divided by the Mie scattering strength that ignores collective scattering at low-ϕ: $[1/\ell^*(\phi)]/[(1/\ell^*_{\mathrm{ISA,Mie}})\phi]$, where $1/\ell^*_{\mathrm{ISA,Mie}} = 0.0207\ \mu\mathrm{m}^{-1}$ is the slope of the calculated $1/\ell^*$ versus ϕ in the dilute limit as $\phi \to 0$, based on independent scattering approximation. As a result, the overall magnitude of the apparent MSDs at different ϕ are reduced by various amounts to obtain the probe self-motion MSDs, as this dimensionless ratio is ϕ-dependent.

To ensure that the number of droplets per scattering volume is time-invariant within during our DWS measurements of concentrateed IA emulsions at higher ϕ, we have also performed Rheolab 3 measurements after only a 10-minute waiting time, which serves as a reference for the reported results at a much longer 24-h waiting time. For all $\phi \geqslant 0.401$, changes in the DWS count rate are less than 3%, and changes in the measured $1/\ell^*$ are less than 4%. Moreover, no distinct layer of cream, which would scatter light more strongly at the top of the cuvette, has been observed visually after the 24-h waiting time. We report dynamic DWS MSD measurements only in the effectively time-invariant range of $\phi \geqslant 0.541$, over which aging and gravity-induced droplet compaction do not significantly influence our measurements and over which the measured DWS $g_2(t)-1$ do not fully decay to baseline in the long-time limit. For $\phi \geqslant 0.64$, a slight decrease in the decay rate of long-time $g_2(t)-1$ beyond $t \approx 2 \times 10^{-1}$ s is observed in measurements after the 24-h waiting time, which is an indication of very slow

aging. This slow aging is not the subject of our study, and our microrheological comparisons are based on plateau MSDs that are at intermediate correlation times, not at long correlation times which show some evidence of slight aging through changes in relaxation. Thus, the primary plateau MSDs, used in the GSER of passive microrheology, occur at shorter times $t \lesssim 2 \times 10^{-2}$ s and are time-invariant over at least 24 h for all $\phi \geqslant 0.541$ that we report. Microrheological comparisons are facilitated by this effective time-invariance of the plateau feature in the MSD over 24-h long waiting time for the elastic dense emulsions. Our purpose in the present study is to make microrheological comparisons of elastic plateau moduli, not to study long-term aging.

Mechanical shear rheometry

We use a 25 mm diameter cone-and-plate geometry (stainless steel) in a controlled-strain mechanical shear rheometer (RFS-II, Rheometric Scientific, equipped with a vapor trap) to measure the plateau elastic shear moduli, $G'_{\mathrm{p,mech}}$, at low strains corresponding to the linear viscoelastic regime. After the same 24-h waiting time also used for DWS, we pre-shear the sample at 50 s^{-1} shear rate for 30 s by stirring with a spatula and load the sample into the rheometer. Measurements are commenced two minutes after lowering the cone to the appropriate pre-set gap with respect to the plate and adjusting the vapor trap. All measurements are performed at $T = 20$ °C. At each ϕ, we perform a small-strain oscillatory frequency sweep from $\omega = 20$ rad/s down to 0.02 rad/s at a small shear strain amplitude of $\gamma = 0.005$. The plateau storage modulus becomes noticeably frequency dependent for $\phi \leqslant 0.620$ at this $\gamma = 0.005$. We next conduct a strain sweep at each ϕ and $\omega = 1$ rad/s, yielding the linear and non-linear shear storage modulus, represented as $G'(\gamma)$. This frequency is within the range of time scales that correspond to the plateau associated with DWS MSD measurements. We probe down to shear strains as low as $\approx 1 \times 10^{-4}$, which is limited by the resolution of the RFS-II's motor. Under small strains, a dominant linear storage modulus can be detected down to $\phi = 0.610$. To obtain the small-strain $G'_{\mathrm{p,mech}}$ of the IA emulsion at each ϕ, we fit the measured $G'(\gamma)$ to a function that has a low-γ-plateau[60] (inset in Fig. 6): $G'(\gamma) = G'_{\mathrm{p,mech}}/[(\gamma/\gamma_{\mathrm{y}})^{\kappa} + 1]$, where γ_{y} is the yield strain associated with the log slope change in $G'(\gamma)$, and κ is a power law exponent related to the non-linear response of $G'(\gamma)$ to larger strains. A two-step yielding strain-response, which has been reported previously in strain sweeps on a similar O/W emulsion system but which had a much stronger attractive strength ($|U_{\mathrm{d}}| \approx 21\ k_{\mathrm{B}}T$)[8], is not apparent in the measured strain sweeps of these IA emulsions.

Regularized fitting using the extended decorated core shell network model

In the prior DCSN model, developed for the SA emulsion, an effective probe-size factor $\alpha_{\mathrm{SA}} = 2.0$, corresponding to a local dense cluster that is approximately tetrahedral on average, has been introduced based on structural concepts for attractive gels of emulsion droplets. Consequently, for attractive colloidal systems, identifying the appropriate probe for interpreting a DWS correlation function as a MSD is complex. The prior study of SA emulsions showed that passive microrheology on these systems can be performed quantitatively via the GSER if the effective size of the DWS scattering probes is taken into account: $G'_{\mathrm{p,GSER}} \propto 1/(\langle a_{\mathrm{pr}}\rangle \langle \Delta \boldsymbol{r}^2\rangle_{\mathrm{p}}) = 1/(\alpha \langle a\rangle \langle \Delta \boldsymbol{r}^2\rangle_{\mathrm{p}})$, where α is the dimensionless ratio between the effective average radius of the DWS scattering probes, $\langle a_{\mathrm{pr}}\rangle$, and the average hydrodynamic radius of an isolated droplet, $\langle a\rangle$. By contrast, the effective DWS probe-size is comparable to $\langle a\rangle$ in the MA system: $\alpha_{\mathrm{MA}} = 1.0$[59]. The IA system, studied herein, has an attractive potential depth that is in between the above-mentioned MA and SA regimes.

To set the magnitude of $\phi_{\mathrm{core,perc}}$ before introducing the fourth principal component of non-percolating droplets for the IA emulsion, we hypothesize an average droplet volume fraction $\phi_{\mathrm{net,core}} = 0.793$ within the percolating core regions, which is the maximum ϕ that we obtained experimentally with the emulsion having this droplet size distribution. We determine G'_{p} within the regions that only have percolating core droplets using the EEI model[27]: $G'_{\mathrm{p,EEI}}(\phi_{\mathrm{net,core}})$. The magnitude of $\phi_{\mathrm{core,perc}}(\phi)$ is determined based on the effective medium assumption: $\phi_{\mathrm{core,perc}}(\phi) = \phi_{\mathrm{net,core}}[G'_{\mathrm{p,GSER}}(\phi)/G'_{\mathrm{p,EEI}}(\phi_{\mathrm{net,core}})]$, where $G'_{\mathrm{p,GSER}}(\phi)$ is determined from the DWS plateau MSD measurements with the assumption of $\alpha = 2$ in the GSER: $G'_{\mathrm{p,GSER}}(\phi) = k_{\mathrm{B}}T/[\pi\alpha\langle a\rangle \langle \Delta \boldsymbol{r}^2(\phi)\rangle_{\mathrm{p}}]$.

Initially, we exclude non-percolating core droplets from consideration, and we minimize χ^2 of the nonlinear least-squares fit for $1/\ell^*_{\mathrm{IA}}(\phi)$ by varying the model's parameters [Eq. (1) with $\phi_{\mathrm{core,nonperc}}$ temporarily set to zero], in a manner similar to what has been previously done for the SA emulsion. This effectively ignores the notch initially but provides a comparable overall shape for the ϕ-dependent functional forms of SDD, shell, and percolating core components. Then, we take into account of $\phi_{\mathrm{core,nonperc}}$ by transferring weights from ϕ_{shell} at $\phi \leqslant 0.64$ and from ϕ_{SDD} within the lower end of notch region. This weight-transfer reflects the reorganization of outer droplets between different clusters as a consequence of the applied shear stress while diluting and mixing. We do so in a manner that preserves the smoothness of all four principal components, even as there are some rapid variations in the notch region itself as SDDs are converted to shell droplets, shell droplets into non-percolating core droplets, and shell and non-percolating core droplets into percolating core droplets. After iterations of minimizing χ^2 of the $1/\ell^*_{\mathrm{IA}}(\phi)$ fit over the entire ϕ range, having all key features considered, we obtain a regularized curve fit of $1/\ell^*_{\mathrm{IA}}(\phi)$ with smooth inter-conversions between all component droplet volume fractions.

Probability density functions of local coordination number

We assume that the distributions for all four principal components in the E-DSCN model are Gaussian; we have also assumed that the same standard deviation of $\sigma_N = 2.5$ is suitable for all of these distributions in order to provide total distributions that are smooth as a function of N for all ϕ considered. Enforcing this smoothness, thus, effectively amounts to a regularization assumption. The peaks of $p_{\mathrm{SDD}}(N)$, $p_{\mathrm{shell}}(N)$, $p_{\mathrm{core,nonperc}}(N)$, and $p_{\mathrm{core,perc}}(N)$ are located at $\langle N \rangle = 3$, 6, 9, and 12, respectively. We emphasize here that these plotted distributions have been inferred, not directly measured. Yet, these distributions show how increasing the osmotic pressure applied to an attractive emulsion can lead to very substantial changes in the local coordination number that are consistent with the measured trends in the optical transport properties of such emulsions over a wide range of ϕ.

Here, we note that $p_N(N \leqslant 2)$ has been folded to larger N, consistent with slippery diffusion-limited cluster aggregation in the dilute limit[16]. In our experimental system, $N = 2$ is theoretically possible, but highly unlikely. Droplets trapped in a mobile bridging configurations, for instance spanning between clusters, can have $N = 2$ in a very unusual situation when clusters are re-established following the cessation of loading and shear disruption. SDDs can also have $N = 2$ in a transient sense if a very strong Brownian excitation breaks one of the three bonds of the SDD and the droplet shifts into a new configuration with only two bonds still present. By contrast, $N \geqslant 3$ represents a relatively stable configuration that is only seldom destabilized by Brownian excitations in IA emulsions, resulting in transient droplet motion on the surface of a cluster rather than complete unbinding and liberation as an isolated droplet. Such transient bound droplet motion is one of the potential sources that could lead to excess DWS MSDs that become particularly noticeable toward lower ϕ.
